# Fatigue and perceived energy in a sample of older adults over 10 years: A resting state functional connectivity study of neural correlates

**DOI:** 10.1016/j.exger.2024.112388

**Published:** 2024-03-02

**Authors:** James B. Hengenius, Rebecca Ehrenkranz, Xiaonan Zhu, Nancy W. Glynn, Theodore J. Huppert, Caterina Rosano

**Affiliations:** a Department of Epidemiology, University of Pittsburgh, Pittsburgh, PA, USA; b Department of Electrical and Computer Engineering, University of Pittsburgh, Pittsburgh, PA, USA

**Keywords:** Self-reported energy, Fatigue, Functional connectivity, Cortico-striatal network

## Abstract

**Purpose:**

Declining energy and increasing fatigue, common in older age, predict neurodegenerative conditions, but their neural substrates are not known. We examined brain resting state connectivity in relation to declining self-reported energy levels (SEL) and occurrence of fatigue over time.

**Methods:**

We examined resting-state functional MRI in 272 community dwelling older adults participating in the Health Aging and Body Composition Study (mean age 83 years; 57.4 % female; 40.8 % Black) with measures of fatigue and SEL collected at regular intervals over the prior ten years. Functional connectivity (FC) between cortex and striatum was examined separately for sensorimotor, executive, and limbic functional subregions. Logistic regression tested the association of FC in each network with prior fatigue state (reporting fatigue at least once or never reporting fatigue), and with SEL decline (divided into stable or declining SEL groups) and adjusted for demographic, physical function, mood, cognition, and comorbidities.

**Results:**

Higher cortico-striatal FC in the right limbic network was associated with lower odds of reporting fatigue (better) at least once during the study period (adjusted odds ratio [95 % confidence interval], *p*-value: (0.747 [0.582, 0.955], 0.020), independent of SEL. Higher cortico-striatal FC in the right executive network was associated with higher odds of declining SEL (worse) during the study period (adjusted odds ratio [95 % confidence interval], *p*-value: (1.31 [1.01, 1.69], 0.041), independent of fatigue. Associations with other networks were not significant.

**Conclusions:**

In this cohort of older adults, the cortico-striatal functional connectivity of declining SEL appears distinct from that underlying fatigue. Studies to further assess the neural correlates of energy and fatigue, and their independent contribution to neurodegenerative conditions are warranted.

## 1. Introduction

Declining self-reported energy level (SEL) and increasing fatigue occur commonly in older age and are correlated with health conditions that are more prevalent in older populations (Cheval et al., 2021; Schwarz et al., 2003). Although they are emerging as predictors of neurodegenerative conditions (Chahine et al., 2021; Tian et al., 2021a), little is known about the neural substrates of SEL and fatigue. Neuroimaging studies have primarily focused on fatigue in neurological patients, indicating an important role for the basal ganglia, and the striatum in particular (Chaudhuri and Behan, 2000; Chaudhuri and Behan, 2004; Friedman and Friedman, 1993). Among PD patients, fatigue was associated with smaller volume of the dorsal striatum (Kluger et al., 2019a), greater task-dependent activation in basal ganglia and frontal cortex (DeLuca et al., 2008), and with higher functional connectivity in limbic and sensorimotor cortex (Tessitore et al., 2016a). One neuroimaging of SEL among cancer patients taking interferon-α, showed higher SEL was associated with higher glucose metabolism in portions of the left striatum (Capuron et al., 2007).

Neuroimaging studies in older adults without neurological disease, albeit sparse and with small sample sizes, support a role for the basal ganglia in fatigue and SEL perception. We found smaller striatum in relation with higher fatigability (Wasson et al., 2019) and lower SEL (Tian et al., 2021b; Ehrenkranz et al., 2021); smaller putamen was associated with higher fatigue and fatigability (Banerjee, 2022; Kluger et al., 2019b).

For the most part, prior studies in older adults without neurological diseases used volumetric measures of gray matter, which are crude indicators of parenchyma integrity, instead of examining markers of network function. Moreover, studies relied on cross-sectional assessments of fatigue and energy, rather than measuring these states repeatedly over time. Fatigue and SEL may be susceptible to variability in stressors; repeated longitudinal measures are more likely to characterize these states stably.

Another limitation of the previous literature on SEL and fatigue is that they are rarely studied independently of each other, and are often considered as one being the opposite of the other. Many scales conflate these two states. For example, scales measuring fatigue ask about energy perception (Berger et al., 2015; Loy et al., 2018a) and vice versa (e.g., asking “During the past 12 months, have you had fatigue or lack of energy more than 3 days?” to measure fatigue) (Cunningham et al., 2015). Yet, there are important differences between fatigue and SEL, indicating each should be studied independently from the other. Work by our group and others in non-clinical populations of older adults indicates that these two states do not completely overlap and that older adults may have declining SEL in the absence of fatigue and vice versa (Ehrenkranz et al., 2023). We have also shown that SEL reflects the capacity to perform physical tasks (e.g., walking faster and performing more physical activity), independent of fatigue and depressive states (Kowalski et al., 2021). Moreover, recent evidence indicates that fatigue is not relieved by common strategies that restore energy (Mota and Pimenta, 2006). Studies of neurobiology support the distinction between SEL and fatigue (Meeusen and Roelands, 2018). Fatigue is primarily driven by the neurotransmitters serotonin and histamine, whereas energy perception is primarily driven by the neurotransmitter dopamine (Loy et al., 2018a; Loy and O’Connor, 2016). However, this evidence is mostly in animal models (Harrington, 2012), young adults (Marcora, 2016), or neurological patients (Chaudhuri and Behan, 2000; Kluger et al., 2016; Barbi et al., 2022).

This study utilized brain resting-state functional connectivity data (functional connectivity describes temporally linked but anatomically separated patterns of neuronal activity) (van den Heuvel and Hulshoff Pol, 2010). We focused specifically on cortico-striatal functional connectivity in the sensorimotor, executive, and limbic cortico-striatal networks. Because of these networks’ role in neurological diseases (for example, Parkinson’s Disease, Mild Parkinsonian Signs, and Huntington’s Disease) (Hengenius et al., 2022; Veldman and Yang, 2018; Tessitore et al., 2016b; O’Callaghan et al., 2014) and in fatigue, motivation, and cognitive function (Haber, 2016; Shepherd, 2013), we investigated the associations between each network with fatigue and SEL.

We hypothesized that higher functional connectivity in these networks would be related to lower fatigue and higher SEL. We leveraged a longitudinal study of aging in a cohort of 272 community-dwelling older adults without neurological disease with repeated measures of fatigue and SEL for 10 years up to the time of the connectivity evaluation.

## 2. Methods

### 2.1. Study population

Participants of the Health Aging and Body Composition Study were recruited from Pittsburgh, Pennsylvania and Memphis, Tennessee in 1997–1998 and were ages 70–79 when enrolled. Of participants who returned for an in-person visit in Pittsburgh in 2006–2008 (ages 79–90), 325 were recruited for the Healthy Brain MRI study. They met the following inclusion criteria: could walk without assistive devices; no hospitalization for major clinic events (physical or psychiatric) in the previous 3 months; no metal present in body; no claustrophobia; less than 250lbs; had mobility measured at their last visit (https://healthabc.nia.nih.gov/, n.d.). Of the 325 participants, 314 underwent 3 T MRI; of these, 42 were excluded due to incomplete scan data, yielding *n* = 272 participants.

Protocols were approved by the University of Pittsburgh institutional review board. Participants provided informed consent at each visit.

### 2.2. Outcomes

Fatigue was captured by the following yes-or-no item: “In the past month, on average, have you been feeling unusually tired during the day?” Data were collected from Years 2–14 of the Healthy Brain study, during which this information was first queried. Participants were categorized as “never fatigued” (answered “no” to the above item for all years on record, *n* = 119) or “fatigued” (answered “yes” to the above item at least once during the course of the Healthy Brain study, *n* = 153). Similar unidimensional tiredness questions to assess fatigue have been used and evaluated previously in population-based research of both healthy and clinical populations and has been shown to be an independent component of fatigue (Mota and Pimenta, 2006; de Vries et al., 2003; Tsuchiya et al., 2016; Jackson, 2015). In sensitivity analyses, we used fatigue burden, i.e., the percentage of visits at which a participant answered “yes” to the above question, to address how fatigue persistence over time relates to FC.

Self-reported energy (SEL) was captured with the following item: “Using this card, please choose the category that best describes your usual energy level in the past month on a scale of 0 to 10 where 0 is no energy and 10 is the most energy that you have ever had.” This question was addressed to participants beginning in year 2 and asked each following year through year 14 (with the exception of year 7). Using these data, energy decline slopes were computed for each participant using linear mixed effect models. Participants were categorized as having “stable energy” (*n* = 94) if their slopes were above the median energy slope for the population (median = −0.0459 subjective energy units/year) (Sprague et al., 2021), or “declining energy” (*n* = 178) if their slopes were below the population median slope. In sensitivity analyses, energy slope was used as continuous rather than binary variable to examine the relationship between energy and FC directly.

### 2.3. Covariates

Age, sex, race, and education (no high school degree or General Educational Diploma, high school completion, post-secondary completion) were collected via self-report in year 1, the time of study entry. All other measures used in the analyses were collected at the time of scan, occurring in years 10–12 of the study. These included body mass index (BMI, kg/m^2^), usual pace gait speed evaluated on a 20 m course, the Modified Mini-Mental State Examination (3MSE) (Teng and Chui, 1987), the digit symbol substitution test (DSST), and the Center for Epidemiologic Studies Depression Scale Revised 10 (CESD-10) (Radloff, 1977).

Measures of cardiovascular and metabolic burden, which could influence fatigue and energy states, included: diabetic status by self-report medication use, fasting plasma glucose >126 mg/dL, or 2-h post-challenge plasma glucose >200 mg/dL; cerebrovascular diseases (including stroke and transient ischemic attack) by self-report and/or medication records; peripheral artery disease via ankle-brachial index below 0.9; hypertension if systolic blood pressure > 140 mmHg or diastolic pressure > 90 mmHg.

As this is a sample of older adults, frailty may affect fatigue and perception of energy. We used the Scale of Aging Vigor in Epidemiology (SAVE), a frailty rating system (0 = no frailty, 10 = most frail) developed by Sanders et al. (Sanders et al., 2011) based on a modified Fried frailty phenotype (FFP) (Fried et al., 2001; Walston et al., 2002). SAVE scores were evaluated at Year 10 (the follow-up visit corresponding to neuroimaging measures in the Health ABC cohort (Wu et al., 2020). A cutoff score ≥ 7 was used to define frail participants (*n* = 36). Due to the small sub-population of frail participants in this sample enriched for healthy individuals, we performed sensitivity analyses excluding frail participants (see Supplemental text).

### 2.4. MRI acquisition

Scanning was conducted at the University of Pittsburgh on a 3T Siemens Magnetom TIM Trio MRI scanner with a Siemens 12-channel head coil. Participants were instructed to remain as still as possible; padding for head and neck reduced head motion. The following sequences were acquired: an axial, whole-brain T1-weighted structural Magnetization Prepared Rapid Acquisition Gradient Echo (MPRAGE) [Repetition Time (TR) = 2300 ms, Echo Time (TE) = 3.43 ms, Flip Angle (FA) =9°, Field of View (FOV) = 224 × 256, 1 mm^3^ isotropic resolution, no acceleration]; and a 5-minute echo-planar imaging sequence (TR = 2000 ms, TE = 34 ms, FA = 90°, FOV = 128 × 128, 2 × 2 × 3 mm resolution) to assess RS blood‑oxygen level dependent response. During RS scanning, no fixation cross or stimulus was presented.

### 2.5. Image processing

Image processing was conducted using Matlab R2014a (MathWorks, Inc., Natick, MA), SPM12 (Wellcome Center for Human Neuroimaging, London, UK), and FSL6.00 (Functional Magnetic Resonance Imaging of the Brain Analysis Group, Oxford, UK). We used SPM for each participant: MPRAGE volumes were segmented into air, soft tissue, skull, cerebrospinal fluid (CSF), gray and white matter (GM, WM) probability maps. Brain masks were defined as the union of the CSF, WM, and GM probability maps after thresholding at 0.1 probability, followed by image filling and closing using Matlab. MPRAGE volumes were warped into Montreal Neurological Institute space using FMRIB’s Nonlinear Image Registration Tool (FNIRT) in FSL.

Slice-timing and motion correction for RS data was performed in SPM (6-DOF search, mutual information cost function) and images were smoothed with an 8 mm FWHM kernel. Skull-stripping was conducted using FSL’s Brain Extraction Tool. Skull-stripped RS volumes were aligned to anatomical volumes using the FMRIB’s Linear Image Registration Tool (FLIRT) with boundary-based registration (using the epi_reg command).

Cortical regions-of-interest (ROIs) were obtained from the Multimodal Parcellation atlas (Glasser et al., 2016) using a volumetric projection of the surface parcellation (https://identifiers.org/neurovault.image:24150), and then categorized as sensorimotor, executive control function (ECF), and limbic regions. Sensorimotor ROIs were defined as Brodmann areas 1, 2, 3a, 3b, 4, 6v, frontal eye fields, premotor eye fields, and supplementary and cingulate eye fields. ECF ROIs were defined as Brodmann areas 46, 9–46d, a9–46v, and p9–46v. Limbic ROIs were defined as Brodmann areas a24 and s32, orbital frontal cortex (OFC), and posterior OFC. Striatal ROIs were obtained from the Oxford- GSK-Imanova Striatal Connectivity atlas (Tziortzi et al., 2014), which divides striatum into sensorimotor, executive, and limbic regions. We considered left and right hemispheres separately.

Prior to connectivity analysis, all ROIs were warped into participant native space using inverse deformation fields from FNIRT. RS time courses were extracted from all ROI voxels, with the first five TRs discarded to account for scanner drift. To address scanner, motion, and physiological noise, we regressed out motion parameters, mean WM signal, mean CSF signal, the first five eigenvariates (accounting for approximately 90 % of the variance) of WM signal after subtracting the mean, and the first five eigenvariates of CSF signal after subtracting the mean.

Mean signal from each cortical functional network was correlated with mean signal from the corresponding striatal region. E.g., to compute sensorimotor cortico-striatal correlation, mean signal from all sensorimotor cortical voxels was correlated with mean signal of sensorimotor striatal voxels. This Pearson correlation was Fisher *Z*-transformed for subsequent statistical analysis.

### 2.6. Statistical analysis

Bivariate associations of participants’ characteristics with fatigue and SEL-change status were examined using independent *t*-tests for continuous variables or chi-square t-tests for categorical variables. Logistic regression models examined whether RS connectivity of each network predicted fatigued/never-fatigued status and SEL-change status. Logistic models were built in two steps to address: 1) adjusted for age; 2) from Step 1, further adjusting for medical risk factors which may be associated with perceived energy and fatigue. Risk factors included systolic blood pressure, BMI, baseline diabetes, and baseline cerebrovascular disease. Sensitivity analyses used linear regression models with continuous measures of fatigue burden (% of visits reporting fatigue) or energy slope, instead of binary indicators. Analyses were conducted in Matlab R2021a (MathWorks, Inc., Natick, MA).

As this is the first study examining these associations and given that our analyses relied on ROIs identified a priori (via literature review to assess biological plausibility), we were more concerned with erroneously ruling out potential findings than with false positives (Rothman, 1990). Accordingly, we did not use multiple comparison correction.

## 3. Results

Participant age ranged from 79 to 90 years (mean ± standard deviation: 83 ± 2.8 years) at the time of MRI. Participants were 40.8 % Black (remainder were White), and 85 % had completed at least a high school education. During the 10 years preceding the time of MRI, 56 % of the sample reported fatigue at least once (range: 1 to up to 4 visits), and 65 % was classified as having reported SEL decline (average decline of 0.47 units over the study time). Among the participants reporting fatigue at least once, 70 % were classified as having declining SEL.

Compared to those never reporting fatigue, participants reporting fatigue at least once were significantly more likely to be women, to have depressive symptoms, and to have cerebrovascular disease (Table 1, all *p <* 0.05). No statistically significant difference was found in other population characteristics, or in relation with SEL decline.

Comparisons of resting-state connectivity by fatigue status, showed that right limbic cortico-striatal connectivity was significantly lower among participants who reported fatigue relative to those who never reported fatigue (between group comparison T-value = −2.34, age adjusted *p* = 0.02). Right sensorimotor cortico-striatal connectivity was lower in participants who reported fatigue relative to those who never reported fatigue, but the difference did not reach statistical significance (between group comparison T-value = −1.65, *p* = 0.0997). Associations with FC of other networks were not significant.

Right executive cortico-striatal connectivity was significantly higher in participants with declining-SEL relative to stable-SEL (between group comparison T-value = 2.065, age-adjusted *p* = 0.04). It should be noted that the two significant cortico-striatal connectivity values (right limbic and right executive cortico-striatal) are not significantly correlated with each other (Pearson’s *r* = 0.0525, *p* = 0.388).

**Fig. 1.**
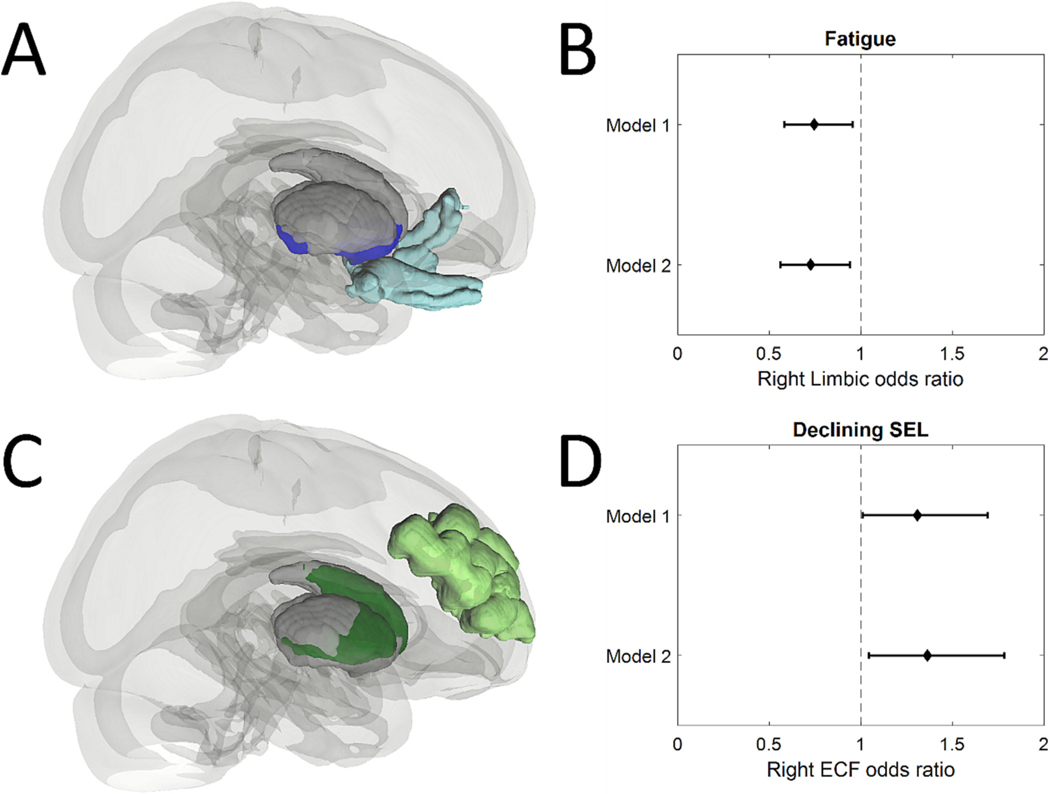
Significant cortico-striatal ROIs and odds ratios for logistic regression. Cortical (light blue) and striatal (dark blue) components of the right limbic cortico-striatal network are shown in (A). Greater connectivity in this network is associated with lower odds of reporting fatigue at least once during the Health ABC study (B). Cortical (light green) and striatal (dark green) components of the right executive cortico-striatal network are shown in (C). Greater connectivity in this network is associated with greater odds of declining SEL during the Health ABC study (D). In plots B and D, Model 1 refers to age-adjusted logistic regression and Model 2 refers to age- and medical risk factor-adjusted odds ratios. (For interpretation of the references to colour in this figure legend, the reader is referred to the web version of this article.)

Associations remained similar when adjusting for other covariates (Table 2). For each unit increase of right limbic cortico-striatal connectivity there was an 18 % lower chance of having reported fatigue at least once (0.726 [0.560, 0.941]). For each unit increase of right executive cortico-striatal connectivity, there was a 36 % higher probability of declining SEL (1.36 [1.04, 1.78]). Network ROIs and adjusted OR values are illustrated in Fig. 1.

Results were similar in sensitivity analyses testing whether network connectivity was associated with SEL slope or fatigue burden using linear regressions and when frail participants were excluded (see supplemental text). Similarly, adjusting for fatigue in the SEL models and adjusting for SEL in the fatigue models does not meaningfully change the results (see supplemental text).

## 4. Discussion

In this cohort of community-dwelling older adults, we find a distinct pattern of cortico-striatal resting state connectivity in relationship to fatigue and energy states. Specifically, reporting fatigue was associated with lower resting state connectivity in the limbic cortico-striatal networks, whereas declining energy was associated with higher resting state connectivity in the executive cortico-striatal network. Higher fatigue and lower energy are both prevalent features of aging in the absence of overt neurological disease (Cheval et al., 2021; Schwarz et al., 2003). However, the neurobiology of these constructs has been primarily studied in populations with neurodegenerative or psychiatric conditions, and the generalizability of findings to healthy aging populations is unclear. As the population of older adults grows, it is increasingly important to investigate the neural correlates underlying their fatigue and energy levels. Targeted therapies to address fatigue have been discovered in PD, yet little has been done to treat fatigue in healthy older adults (Kelberman and Vazey, 2016). Addressing this gap in the neurobiology of fatigue and energy lays the groundwork for future targeted clinical interventions in older adults.

We find lower limbic cortico-striatal connectivity was associated with increasing odds of having reported fatigue at least once in prior years. Our findings parallel prior neuroimaging work in some clinical populations, suggesting that the neural substrates implicated in pathology-related fatigue may also be important for fatigue regulation in adults without neurological diseases. The striatum is implicated in the experience of fatigue in PD (Kluger et al., 2019a); functional differences in sensorimotor and limbic ROIs were implicated in fatigued vs not-fatigued groups (Tessitore et al., 2016a). Hypoperfusion of the limbic system (including putamen, anterior cingulate cortex, and pallidum) has been associated with myalgic encephalitis/chronic fatigue syndrome (Li et al., 2021).

SEL is less studied than fatigue in the neuroimaging literature, but executive cortical and striatal regions have been implicated in the perception of energy and wellbeing (Boniwell and Zimbardo, 2015). For example, one electroencephalography study of healthy adults implicated executive control function networks in SEL perception (Wyczesany et al., 2011). In cancer patients, higher cross-sectional SEL was associated with higher glucose metabolism in portions of the left striatum (Capuron et al., 2007). In healthy older adults from the cohort examined in this work, decline in SEL was also associated with lower striatal volume (Tian et al., 2021b). In a task-induced fatigue study that measured the degree of being rested using a visual analogue scale, decreased frontoparietal network activity was detected (Esposito et al., 2014). In a resting-state study of young adults, a negative correlation was found between connectivity in the dorsolateral prefrontal cortex (Brodmann areas 9 and 46) and wellbeing was found (Chen et al., 2022).

Because nigrostriatal dopaminergic signaling is associated with higher SEL (Loy et al., 2018b), and cortico-striatal networks are thought to rely on dopaminergic signaling, we hypothesized that greater functional connectivity in cortico-striatal networks would be associated with reduced odds of declining SEL. However, we found the opposite. The existing literature on corticostriatal connectivity and measures associated with wellbeing (such as SEL) is limited and the direction of association inconsistent (de Vries et al., 2023). This may in part be due to different study populations, primarily those with psychiatric disorders and/or cognitive impairment and adults under age 40 (de Vries et al., 2023). Interestingly, a study of fatigue in PD noted higher functional connectivity within lateral prefrontal cortex among fatigued patients (Tessitore et al., 2016a).

There are limitations to this work. While longitudinal measures of fatigue and SEL were available over a relatively long period of time, neuroimaging data was available from only a single timepoint, at the end of follow-up. Thus, we cannot determine whether changes in neuroimaging markers precede or follow changes in energy and fatigue levels. The assessment of SEL relied on one single question. Other instruments that differentiate between dimensions of fatigue and SEL should be used in future studies (E.g., the Lee Scale, which frames fatigue in terms of drowsiness, fatigue, exhaustion, and SEL in terms of energy and efficiency) (Lerdal et al., 2013). Likewise, our binary measure of fatigue as tiredness does not capture dimensionality in fatigue, limiting our ability to probe different trajectories of fatigue. Fatigue itself is a multidimensional construct including physical and mental domains, which our participant questions do not distinguish. Perceived fatigue is also related to participants’ regular activity levels, which is not assessed here. An alternative to fatigue inventory measures is fatigability, a measure of perceived fatigue normalized to a particular task or activity (Eldadah, 2010). Previous work by our group has revealed volumetric changes in striatal and other structures related to fatigability in healthy populations (Wasson et al., 2019), and adopting this approach in future work may allow for more sensitivity in functional neuroimaging analyses than our coarse measure of all-or-nothing fatigue (Simonsick et al., 2018).

This protocol did not include field maps, so uncorrected susceptibility distortions may lead to increased noise and false negatives in anterio-ventral limbic ROIs. Finally, the resting-state protocol was five minutes; subsequent studies have suggested longer RS scans can reduce variability in network inference, though five minutes is sufficient for accurate connectivity inference (Birn et al., 2013).

One strength of this work is its focus on older adults without neurological conditions. While neuroimaging studies of fatigue and SEL have focused on clinical populations, our studies may better generalize at the population level. Building on our past work with this cohort, our approach of distinguishing SEL from fatigue is novel and has revealed differing neural substrates for each construct. Though minorities are often underrepresented in neuroimaging studies, the relatively high percentage of Black participants in this cohort allows greater generalizability of results.

This work suggests there are distinct cortico-striatal bases for SEL and energy. Future neuroimaging investigations of energy and fatigue should distinguish between these two constructs rather than treating them as two ends of a continuum. As in PD, understanding the neurobiology may be the initial step towards targeted and effective interventions.

## Supplementary Material

1

## Figures and Tables

**Table 1:** Sample demographic, function, and comorbidities.

		All cohort	Fatigue^^^		Energy^^^^	
			Never	At least once	Stable	Declining
Number (%)		272	119 (43.8)	153 (56.3)	94 (34.6)	178 (65.4)
Demographic	Age, years	82.97 ± 2.77	83.03 ± 2.59	82.94 ± 2.91	82.93 ± 2.71	83.01 ± 2.81
	Women	156 (57.4)	**57 (47.9)**	**99 (64.7)**	59 (62.8)	97 (54.5)
	Black	111 (40.8)	51 (42.9)	60 (39.2)	43 (45.7)	68 (38.2)
	Education≥ HS,	231 (84.9)	103 (86.6)	128 (83.7)	80 (85.1)	151 (84.8)
Overall function	Modified Minimental, 0–100	93.11 ± 6.61	93.50 ± 6.08	92.80 ± 7.01	92.67 ± 6.69	93.34 ± 6.58
	Digit Symbol Substitution Test	36.81 ± 13.51	37.24 ± 14.72	36.47 ± 12.49	37.01 ± 13.35	36.71 ± 13.62
	Center for Epidemiologic Studies Depression Scale, 0–30	7.00 ± 5.99	**4.86 ± 4.54**	**8.75 ± 6.46**	6.32 ± 5.70	7.38 ± 6.13
	Gait speed, m/s	1.00 ± 0.28	1.02 ± 0.30	0.99 ± 0.28	1.01 ± 0.27	1.00 ± 0.29
	SEL slope, subjective energy units/year	− 0.047 ± 0.026	− 0.041 ± 0.023	− 0.052 ± 0.027	− 0.027 ± 0.019	− 0.058 ± 0.023
	Fraction of visits reporting fatigue	0.240 ± 0.280	–	0.422 ± 0.250	0.199 ± 0.264	0.261 ± 0.285
Comorbidities	Systolic blood pressure, mmHG	134.88 ± 19.31	134.82 ± 19.13	134.93 ± 19.52	133.34 ± 19.69	135.69 ± 19.12
	BMI, kg/m^2^	27.48 ± 4.42	27.11 ± 4.54	27.78 ± 4.31	27.12 ± 4.00	27.68 ± 4.62
	Diabetes, present	70 (25.7)	26 (21.9)	44 (28.8)	20 (21.3)	50 (28.1)
	Cerebrovascular disease, present	10 (3.7)	**1 (0.9)**	**9 (5.9)**	3 (3.2)	7 (4.0)
	Peripheral arterial disease, present	49 (20.8)	21 (20.6)	28 (20.9)	15 (17.4)	34 (22.7)

Continuous variables reported as mean ± SD.

Categorical variables reported as N (&).

SEL, self-reported energy level; BMI, body mass index.

^ Participants were classified as fatigued if they reported fatigue at least once during the study follow-up and were classified a never-fatigued if they never reported fatigue during follow-up.

^^ Participants were classified as having stable energy if their SEL slope was greater than one standard deviation below the sample mean slope and were classified as having declining energy if their SEL slope was less than one standard deviation below the population mean slope.

Bold values indicate significant differences (*p <* 0.05) as indicated by *t*-test (continuous) or chi-squared test (categorical).

**Table 2:** Logistic regression models of fatigue and SEL onto connectivity.

	Age-adjusted	Further adjusted for medical risk factors^a^
Outcome: fatigued at least once	Odds ratio [95 % CI], *p*-value	
RS connectivity of the right limbic network	0.747 [0.582, 0.955], 0.020	0.726 [0.560, 0.941], 0.016
RS connectivity of the right executive network	1.22 [0.561, 2.65], 0.799	1.03 [0.469, 2.28], 0.967
Outcome: declining-SEL	Odds ratio [95 % CI] p-value	
RS connectivity of the right limbic network	1.11 [0.574, 2.13], 0.878	1.13 [0.584, 2.20], 0.852
RS connectivity of the right executive network	1.31 [1.01, 1.69], 0.041	1.36 [1.04, 1.78], 0.023

a Medical risk factors include systolic blood pressure, body mass index, baseline diabetes, baseline cerebrovascular disease.

## Data Availability

Data will be made available on request.
