# Supplementary material for: Fatigue and perceived energy in a sample of older adults over 10 years: A resting state functional connectivity study of neural correlates
Source: Exp Gerontol. Author manuscript; Available in PMC 2024 Apr 22. (PMC11033705; doi:10.1016/j.exger.2024.112388)
Supplement: 1 [file NIHMS1979579-supplement-1.docx]

# **Supplement 1: Sensitivity Analyses for “Changes in fatigue and perceived energy in older adults: a functional connectivity study of cortico-striatal networks.”**

As discussed in Methods of the main text, we performed sensitivity analyses to compare the results of logistic regression models using binary outcomes (“never fatigued” vs. “fatigued” and “declining energy” vs. “stable energy”) with results of linear regression models using continuous outcomes (proportion of follow-up visits in which participants reported feeling fatigued and longitudinal self-reported energy slopes). We also compared our main findings with models excluding participants considered frail (SAVE scores ≥ 7) as evaluated at the time of MRI scanning (year 10 of Health ABC). Sensitivity models and results follow.

## Continuous Fatigue Model

Rather than categorizing participants as “never fatigued” (never reporting fatigue during follow-up visits) or “fatigued” (reporting fatigue in at least one follow-up visit), we considered the fraction of visits in which participants reported fatigue, *F_fatigue_*, and performed linear regression against corticostriatal connectivity variables within each hemisphere of the brain, adjusting first for age and then for age plus additional medical covariates (systolic blood pressure, body mass index, baseline diabetes, baseline, and cerebrovascular disease).

The forms of the models in Wilkinson notation are,

*F_fatigue_* ~ 1 + *Connectivity* + *Age* (Model 1)

*F_fatigue_* ~ 1 + *Connectivity* + *Age* + *BP_systolic_* + *BMI* + *Diabetes_baseline_* + *CBVD_baseline_*. (Model 2)

Here, *Connectivity* refers to corticostriatal functional connectivity between the ECF cortical network and striatum in the left or right hemisphere, corticostriatal functional connectivity between the somatomotor cortical network and striatum in the left or right hemisphere, or corticostriatal functional connectivity between the limbic cortical network and striatum in the left or right hemisphere. *Age* is the participant age at time of MRI scan, *BP_­systolic_* is the systolic blood pressure at time of scan, *BMI* is body mass index, *Diabetes_baseline_* is the presence of absence of a diabetes diagnosis at entry into the study, and *CBVD_baseline_* is the presence of a cerebrovascular disease diagnosis at time of entry into the study.

Model results for each corticostriatal connectivity measure with continuous fatigue fraction are shown in **Table S1**. Use of a continuous fatigue metric in the age-adjusted model reduces the association of right limbic corticostriatal connectivity from significant to suggestive (p<0.05 to p<0.10), but inclusion of additional medical covariates in the model indicates a significant association between right limbic corticostriatal connectivity and fatigue (p<0.05).

## Continuous Self-Reported Energy Level (SEL) Decline Model

Rather than categorizing participants as “declining energy” (longitudinal slope of SEL below the median value) or “stable energy” (longitudinal slope of SEL above the median value), we considered the slope itself, *S_SEL_*, and performed linear regression against corticostriatal connectivity variables within each hemisphere of the brain, adjusting first for age and then for age plus additional medical covariates (systolic blood pressure, body mass index, baseline diabetes, baseline, and cerebrovascular disease).

The forms of the models in Wilkinson notation are,

*S_SEL_* ~ 1 + *Connectivity* + *Age* (Model 1)

*S_SEL_* ~ 1 + *Connectivity* + *Age* + *BP_systolic_* + *BMI* + *Diabetes_baseline_* + *CBVD_baseline_*. (Model 2)

Independent model terms are identical to those described above.

Model results for each corticostriatal connectivity measure with continuous SEL slope are shown in **Table S2**. Use of a continuous SEL metric in the age-adjusted model shows no significant nor suggestive association of right ECF corticostriatal connectivity (p>0.1), though the direction of association is maintained (higher functional connectivity indicates more negative SEL slopes and greater decline in energy). Addition of medical covariates further shows similar non-significant model behavior.

## Binary Fatigue Model Excluding Frail Participants

Returning to the binary outcomes of “never fatigued” (never reporting fatigue over duration of follow-up, *Fatigue* = 0) and “fatigued” (reporting fatigue in at least one follow-up visit, *Fatigue* = 1), we examined how exclusion of frail participants (with Scale of Aging Vigor in Epidemiology (SAVE) frailty scores ≥7 on a scale of 0 to 10, measured in year 10 of follow-up so as to be as near as possible to the time of MRI scan) affected the association of corticostriatal connectivity variables within each hemisphere and the fatigue outcome. Models are adjusted first for age and then for age plus additional medical covariates (systolic blood pressure, body mass index, baseline diabetes, baseline, and cerebrovascular disease).

The forms of the models in Wilkinson notation are,

*Fatigue* ~ 1 + *Connectivity* + *Age* (Model 1)

*Fatigue* ~ 1 + *Connectivity* + *Age* + *BP_systolic_* + *BMI* + *Diabetes_baseline_* + *CBVD_baseline_*. (Model 2)

Independent model terms are identical to those described above.

Model results for each corticostriatal connectivity measure with the binary *Fatigue* variable are shown in **Table S3**. Exclusion of frail participants in the age-adjusted model shows no effect on the significance of right limbic corticostriatal connectivity (p remains <0.05). The addition of medical covariates shows similar model behavior.

## Self-Reported Energy Level (SEL) Decline Model Excluding Frail Participants

Returning to the binary outcomes of “declining energy” (longitudinal slope of SEL below the median value, *SEL_decline_* = 1) and “stable energy” (longitudinal slope of SEL above the median value, *SEL_decline_* = 0), we examined how exclusion of frail participants affected the association of corticostriatal connectivity variables within each hemisphere and the SEL decline outcome. Models are adjusted first for age and then for age plus additional medical covariates (systolic blood pressure, body mass index, baseline diabetes, baseline, and cerebrovascular disease).

The forms of the models in Wilkinson notation are,

*SEL_decline_* ~ 1 + *Connectivity* + *Age* (Model 1)

*SEL_decline_* ~ 1 + *Connectivity* + *Age* + *BP_systolic_* + *BMI* + *Diabetes_baseline_* + *CBVD_baseline_*. (Model 2)

Independent model terms are identical to those described above.

Model results for each corticostriatal connectivity measure with discretized *SEL_decline_* variable are shown in **Table S4**. Exclusion of frail participants in the age-adjusted model shows no effect on the significance of right ECF corticostriatal connectivity (p remains <0.05). The addition of medical covariates shows similar model behavior.

## Binary Fatigue Model Adjusting for SEL Decline Status

To address whether fatigue status is associated with SEL decline status (as may be expected if the two measures reflect an underlying fatigue/energy construct, we included the binary SEL decline measure as a covariate in our models of binary fatigue status regressed onto connectivity. This analysis was performed with and without adjustment for medical covariates.

The forms of the models in Wilkinson notation are,

*Fatigue* ~ 1 + *Connectivity* + *Age* + *SEL_decline_* (Model 1)

*Fatigue* ~ 1 + *Connectivity* + *Age* + *BP_systolic_* + *BMI* + *Diabetes_baseline_* + *CBVD_baseline_* + *SEL_decline_*. (Model 2)

Model terms are identical to those described above.

Model results for each corticostriatal connectivity measure with the binary *Fatigue* variable are shown in **Table S5**. Inclusion of SEL decline status in the age-adjusted model and the additionally medical covariate-adjusted model shows no effect on the significance of right limbic corticostriatal connectivity (p remains <0.05).

## Binary SEL Decline Model Adjusting for Never-Fatigued Status

To consider the moderating effect of fatigue status on the relationship of functional connectivity and SEL decline status, we included the binary fatigue measure as a covariate in our models of binary SEL decline status regressed onto connectivity. This analysis was performed with and without adjustment for medical covariates.

The forms of the models in Wilkinson notation are,

*SEL_decline_* ~ 1 + *Connectivity* + *Age* + *Fatigue* (Model 1)

*SEL_decline_* ~ 1 + *Connectivity* + *Age* + *BP_systolic_* + *BMI* + *Diabetes_baseline_* + *CBVD_baseline_* + *Fatigue*. (Model 2)

Model terms are identical to those described above.

Model results for each corticostriatal connectivity measure with the binary *SEL_decline_* variable are shown in **Table S6**. Inclusion of fatigue status in the age-adjusted model and the additionally medical covariate-adjusted model shows no effect on the significance of right ECF corticostriatal connectivity (p remains <0.05).

**Table S1: Associations between corticostriatal connectivity and proportion of visits reporting fatigue**

| Corticostriatal network measure (hemisphere) | Standardized Effect size  (95% CI) | p-value |  |
| --- | --- | --- | --- |
| Model 1 (Age-adjusted) | | | |
| SM (left) | -0.002 (-0.122, 0.117) | 0.97 |  |
| SM (right) | 0.074 (-0.046, 0.193) | 0.23 |  |
| ECF (left) | -0.024 (-0.143, 0.096) | 0.70 |  |
| ECF (right) | 0.037 (-0.082, 0.157) | 0.54 |  |
| LM (left) | 0.02 (-0.095, 0.145) | 0.68 |  |
| LM (right) | -0.108 (-0.227, 0.0115) | 0.08 |  |
| Model 2 (Age- and medical covariate-adjusted*) | | | |
| SM (left) | -0.003 (-0.12,0.114) | 0.96 |  |
| SM (right) | 0.068 (-0.051,0.187) | 0.26 |  |
| ECF (left) | -0.043 (-0.161,0.075) | 0.48 |  |
| ECF (right) | 0.001 (-0.118,0.12) | 0.99 |  |
| LM (left) | -0.017 (-0.139,0.106) | 0.79 |  |
| LM (right) | -0.122 (-0.239, -0.004) | **0.04** |  |

* Medical covariates are systolic blood pressure, body mass index, baseline diabetes, baseline, and cerebrovascular disease

**Table S2: Associations between corticostriatal connectivity and SEL slope**

| Corticostriatal network measure  (hemisphere) | Standardized Effect size  (95% CI) | p-value |  |
| --- | --- | --- | --- |
| Model 1 (Age-adjusted) | | | |
| SM (left) | 0.072 (-0.049, 0.192) | 0.24 |  |
| SM (right) | 0.034 (-0.086, 0.155) | 0.58 |  |
| ECF (left) | -0.038 (-0.160, 0.084) | 0.54 |  |
| ECF (right) | -0.085 (-0.205, 0.035) | 0.17 |  |
| LM (left) | -0.053 (-0.175, 0.068) | 0.39 |  |
| LM (right) | 0.037 (-0.085, 0.158) | 0.55 |  |
| Model 2 (Age- and medical covariate-adjusted) | | | |
| SM (left) | 0.066 (-0.054, 0.187) | 0.28 |  |
| SM (right) | 0.049 (-0.073, 0.172) | 0.43 |  |
| ECF (left) | -0.031 (-0.155, 0.092) | 0.62 |  |
| ECF (right) | -0.078 (-0.200, 0.045) | 0.22 |  |
| LM (left) | -0.040 (-0.168, 0.087) | 0.54 |  |
| LM (right) | 0.038 (-0.084, 0.161) | 0.54 |  |

* Medical covariates are systolic blood pressure, body mass index, baseline diabetes, baseline, and cerebrovascular disease

**Table S3: Associations between corticostriatal connectivity and fatigue status, excluding frail individuals**

| Corticostriatal network measure  (hemisphere) | Odds ratio  (95% CI) | p-value |  |
| --- | --- | --- | --- |
| Model 1 (Age-adjusted) | | | |
| SM (left) | 0.956 (0.752, 1.216) | 0.71 |  |
| SM (right) | 1.231 (0.964, 1.572) | 0.10 |  |
| ECF (left) | 0.980 (0.771, 1.246) | 0.87 |  |
| ECF (right) | 1.032 (0.811, 1.312) | 0.80 |  |
| LM (left) | 0.992 (0.78, 1.262) | 0.95 |  |
| LM (right) | 0.746 (0.582, 0.955) | **0.02** |  |
| Model 2 (Age- and medical covariate-adjusted) | | | |
| SM (left) | 0.932 (0.718, 1.209) | 0.60 |  |
| SM (right) | 1.219 (0.932, 1.593) | 0.15 |  |
| ECF (left) | 0.970 (0.746, 1.262) | 0.82 |  |
| ECF (right) | 1.049 (0.808, 1.362) | 0.72 |  |
| LM (left) | 0.937 (0.717, 1.226) | 0.64 |  |
| LM (right) | 0.735 (0.56, 0.966) | **0.03** |  |

* Medical covariates are systolic blood pressure, body mass index, baseline diabetes, baseline, and cerebrovascular disease

**Table S4: Associations between corticostriatal connectivity and SEL decline status, excluding frail individuals**

| Corticostriatal network measure  (hemisphere) | Odds ratio  (95% CI) | p-value |  |
| --- | --- | --- | --- |
| Model 1 (Age-adjusted) | | | |
| SM (left) | 0.908 (0.696, 1.185) | 0.48 |  |
| SM (right) | 0.993 (0.762, 1.294) | 0.96 |  |
| ECF (left) | 0.931 (0.715, 1.213) | 0.60 |  |
| ECF (right) | 1.407 (1.065, 1.858) | **0.02** |  |
| LM (left) | 1.061 (0.813, 1.384) | 0.66 |  |
| LM (right) | 1.003 (0.77, 1.306) | 0.98 |  |
| Model 2 (Age- and medical covariate-adjusted) | | | |
| SM (left) | 0.913 (0.699, 1.192) | 0.50 |  |
| SM (right) | 1.002 (0.765, 1.312) | 0.99 |  |
| ECF (left) | 0.940 (0.72, 1.228) | 0.65 |  |
| ECF (right) | 1.418 (1.072, 1.877) | **0.01** |  |
| LM (left) | 1.076 (0.818, 1.415) | 0.60 |  |
| LM (right) | 1.006 (0.77, 1.315) | 0.96 |  |

* Medical covariates are systolic blood pressure, body mass index, baseline diabetes, baseline, and cerebrovascular disease

**Table S5: Associations between corticostriatal connectivity and fatigue status, adjusting for SEL decline status**

| Corticostriatal network measure  (hemisphere) | Odds ratio  (95% CI) | p-value |  |
| --- | --- | --- | --- |
| Model 1 (Age-adjusted) | | | |
| SM (left) | 0.971 (0.762, 1.239) | 0.81 |  |
| SM (right) | 1.23 (0.961, 1.575) | 0.10 |  |
| ECF (left) | 0.993 (0.779, 1.266) | 0.95 |  |
| ECF (right) | 0.996 (0.78, 1.271) | 0.97 |  |
| LM (left) | 0.981 (0.77, 1.251) | 0.88 |  |
| LM (right) | **0.739 (0.576, 0.95)** | **0.02** |  |
| Model 2 (Age- and medical covariate-adjusted) | | | |
| SM (left) | 0.971 (0.759, 1.242) | 0.82 |  |
| SM (right) | 1.242 (0.961, 1.605) | 0.10 |  |
| ECF (left) | 0.972 (0.756, 1.248) | 0.82 |  |
| ECF (right) | 0.955 (0.743, 1.228) | 0.72 |  |
| LM (left) | 0.938 (0.726, 1.213) | 0.63 |  |
| LM (right) | **0.719 (0.554, 0.931)** | **0.01** |  |

* Medical covariates are systolic blood pressure, body mass index, baseline diabetes, baseline, and cerebrovascular disease

**Table S6: Associations between corticostriatal connectivity and SEL decline status, adjusting for fatigue status**

| Corticostriatal network measure  (hemisphere) | Odds ratio  (95% CI) | p-value |  |
| --- | --- | --- | --- |
| Model 1 (Age-adjusted) | | | |
| SM (left) | 0.885 (0.684, 1.144) | 0.35 |  |
| SM (right) | 1.004 (0.778, 1.295) | 0.98 |  |
| ECF (left) | 0.907 (0.704, 1.17) | 0.45 |  |
| ECF (right) | **1.313 (1.011, 1.705)** | **0.04** |  |
| LM (left) | 1.087 (0.841, 1.404) | 0.52 |  |
| LM (right) | 1.066 (0.825, 1.378) | 0.63 |  |
| Model 2 (Age- and medical covariate-adjusted) | | | |
| SM (left) | 0.891 (0.688, 1.153) | 0.38 |  |
| SM (right) | 0.969 (0.746, 1.258) | 0.81 |  |
| ECF (left) | 0.909 (0.703, 1.177) | 0.47 |  |
| ECF (right) | **1.345 (1.027, 1.761)** | **0.03** |  |
| LM (left) | 1.103 (0.843, 1.445) | 0.47 |  |
| LM (right) | 1.069 (0.823, 1.388) | 0.62 |  |

* Medical covariates are systolic blood pressure, body mass index, baseline diabetes, baseline, and cerebrovascular disease
